# What view information is most important in the homeward navigation of an Australian bull ant, *Myrmecia midas*?

**DOI:** 10.1007/s00359-022-01565-y

**Published:** 2022-09-01

**Authors:** Muzahid Islam, Sudhakar Deeti, Trevor Murray, Ken Cheng

**Affiliations:** School of Natural Sciences, Macquarie University, Sydney, NSW 2109 Australia

**Keywords:** Terrestrial panorama, Celestial cue, Heading direction, Homeward navigation, Familiar environment

## Abstract

Many insects orient by comparing current panoramic views of their environment to memorised views. We tested the navigational abilities of night-active *Myrmecia midas* foragers while we blocked segments of their visual panorama. Foragers failed to orient homewards when the front view, lower elevations, entire terrestrial surround, or the full panorama was blocked. Initial scanning increased whenever the visual panorama was blocked but scanning only increased along the rest of the route when the front, back, higher, or lower elevations were blocked. Ants meandered more when the front, the back, or the higher elevations were obscured. When everything except the canopy was blocked, the ants were quick and direct, but moved in random directions, as if to escape. We conclude that a clear front view, or a clear lower panorama is necessary for initial homeward headings. Furthermore, the canopy is neither necessary nor sufficient for homeward initial heading, and the back and upper segments of views, while not necessary, do make finding home easier. Discrepancies between image analysis and ant behaviour when the upper and lower views were blocked suggests that ants are selective in what portions of the scene they attend to or learn.

**Supplementary information:**

The online version contains supplementary material available at 10.1007/s00359-022-01565-y.

## Introduction

Individually foraging ants navigate using the terrestrial panorama and path integration for their visual navigation (Wehner 2009, 2020; Cheng 2012; Collett et al. 2014; Freas and Schultheiss 2018). Foragers acquire and retain information from the surrounding terrestrial panorama to navigate between the nest and foraging sites when visual information is available Collett et al. 2006; Collett 2010; Mangan and Webb 2012; Freas et al. 2017a; Islam et al. 2020; Wehner 2020; Islam et al. 2021). A naive ant acquires views of the terrestrial panorama around its nest through a series of learning walks before it begins foraging and uses these learned views for navigation later in life (Zeil 2012; Fleischmann et al. 2016, 2017; Jayatilaka et al. 2018; Freas et al. 2019; Zeil and Fleischmann 2019; Deeti and Cheng 2021). In path integration, insects combine odometric information, to track the distance travelled, with the use of multiple celestial cues, including the position of the sun or moon and the pattern of polarised light in the sky, for estimating compass direction (Wehner and Müller 2006; Wehner 2009; Freas et al. 2017b; Buehlmann et al. 2018; Heinze et al. 2018; Webb 2019). Ants rely most on celestial information when the environment lacks terrestrial information or when foragers are inexperienced or unfamiliar with the local panorama (Bühlmann et al. 2011; Freas and Cheng 2017; Freas and Cheng 2019; review: Cheng et al. 2014). Although ants living in different habitats favour the use of celestial vs. terrestrial cues differently (Cheng et al. 2014), most ants use both kinds of information for navigation (Wehner 2003; Cheng et al. 2014), including the study species of the current account, *Myrmecia midas* (Freas et al. 2017a, b).

With regard to the use of terrestrial cues, foraging ants develop robust memories of foraging sites and navigational routes by learning the surrounding panorama (Wehner et al. 1996; Kohler and Wehner 2005; Graham and Cheng 2009; Mangan and Webb 2012; Zeil 2012; Schultheiss et al. 2016; Freas and Cheng 2018; Freas et al. 2019; Freas and Spetch 2019). Besides the aforementioned learning walks, foragers also turn back to their nest direction occasionally as they venture forth farther and farther in foraging, oscillating towards and then away from their nest (Freas and Cheng 2018; Zeil and Fleischmann 2019). Foragers compare the current view with their memorised views of the nest-directed or route-directed panoramas to return to their nest (Collett et al. 2006; Wehner et al. 2006; Wystrach et al. 2011; Wystrach et al. 2012; Narendra et al. 2013; Zeil et al. 2014; Stürzl and Zeil 2007; modelling: Philippides et al. 2011; Baddeley et al. 2012; Zeil 2012; Ardin et al. 2016). Each learned view has a catchment area: the area over which that image provides enough navigational information for a forager to return to the location of the learned view. The catchment area depends in good part on how visually cluttered the environment is (Stürzl and Zeil 2007; Narendra et al. 2013; Murray and Zeil 2017). The navigational success of foraging ants in an environment depends on the scene similarity between the release-site panorama and previously acquired panoramic information (Freas and Cheng 2019; Freas et al. 2019; Deeti et al. 2020; Islam et al. 2021a).

Several diurnal ants (Fukushi 2001; Fukushi and Wehner 2004; Graham and Cheng 2009; Wystrach et al. 2011) and nocturnal ants (Reid et al. 2011; Narendra et al. 2013; Freas et al. 2019) use the terrestrial panorama to determine the direction for homeward navigation (Zeil et al. 2014; Freas et al. 2019). In *Myrmecia pyriformis*, the front and back panoramic views were especially important for determining heading direction compared to the left-side or right-side view (Reid et al. 2011). In the Australian desert ants, *Melophorus bagoti*, a simple approximation of the terrestrial panorama is sufficient for orienting towards the nest direction, and their ability to orient is robust to changes in elevation, even when close-by objects dominate large portions of the scene (Graham and Cheng 2009; Schwarz et al. 2014). In both *Myrmecia* and *Melophorus* species, when the panorama in the direction of heading is obscured or was unfamiliar, foragers take longer to travel, were less directed, and were sometimes unable to move towards the goal (Reid et al. 2011; Wystrach et al. 2012, 2014). Other experiments showed that when the skyline in a certain direction (e.g., to the left) was higher than the height of the ants’ remembered view, that biased the ants’ initial orientation away from the portion that looked too high (Graham and Collett 2002; Wystrach et al. 2012; Julle-Daniere et al. 2014). Already reviewed, *M. midas* navigate using both the terrestrial visual panorama and celestial cues in their natural environment. Freas et al. 2017a, b, 2018; Freas and Cheng 2019; Islam et al. 2020; Islam et al. 2021a; Islam et al. 2021b). Which portions of view information are more crucial for navigation in this nocturnal ant has not yet been studied, and we now test this question comprehensively.

*Myrmecia midas* furnishes a crucial datapoint in understanding the extent to which visual scene clutter contributes to navigational strategies. It inhabits a dense visual environment, forages in dim twilight, and yet is a competent vision-based navigator. Here we explore the extent to which these ants utilise different portions of the visual panorama for navigation by blocking view segments at a familiar location during a homing trip. We test their reliance on path integration and terrestrial-panorama-based navigation by blocking either the celestial cues or portions of the terrestrial panorama. We further investigate view learning and attention, by blocking specific segments of a terrestrial panorama and comparing the effect of their absence on navigation. Together, these results should give us a clearer understanding of how differences in visual environments shape navigational strategies, evolutionarily, behaviourally, and neurobiologically.

## Materials and methods

### Study species

Our view-blocking experiments were conducted on the nocturnal bull ant, *Myrmecia midas*, from March to April 2020 on the Macquarie University, North Ryde campus in Sydney, Australia (33°46′18″ S, 151°06′30″ E). Two nests were selected ∼ 25 m apart, where foragers navigated to nearby trees within a 12 m radius of the nest. The nests of *M. midas* at this field site were found in wooded areas with stands of eucalyptus trees and with the ground mostly covered with bark, wood chips, leaf litter, and small grasses. Like most *M. midas* nests, these nests were located at the base of eucalyptus trees, which many of that nest’s ants forage on, and which we call nest trees. The remaining foragers travel to more distant surrounding trees, which we call foraging trees. *M. midas* foragers have high tree fidelity and depart to forage in the evening twilight and return as late as the morning twilight (Freas et al. 2017a). While researching and collecting ants requires no ethical approval at the local or national level in Australia, we took care to ensure all ants were returned to their nests, and that our experimental procedures had no adverse effects on individuals or the colonies as a whole.

**Fig. 1:**
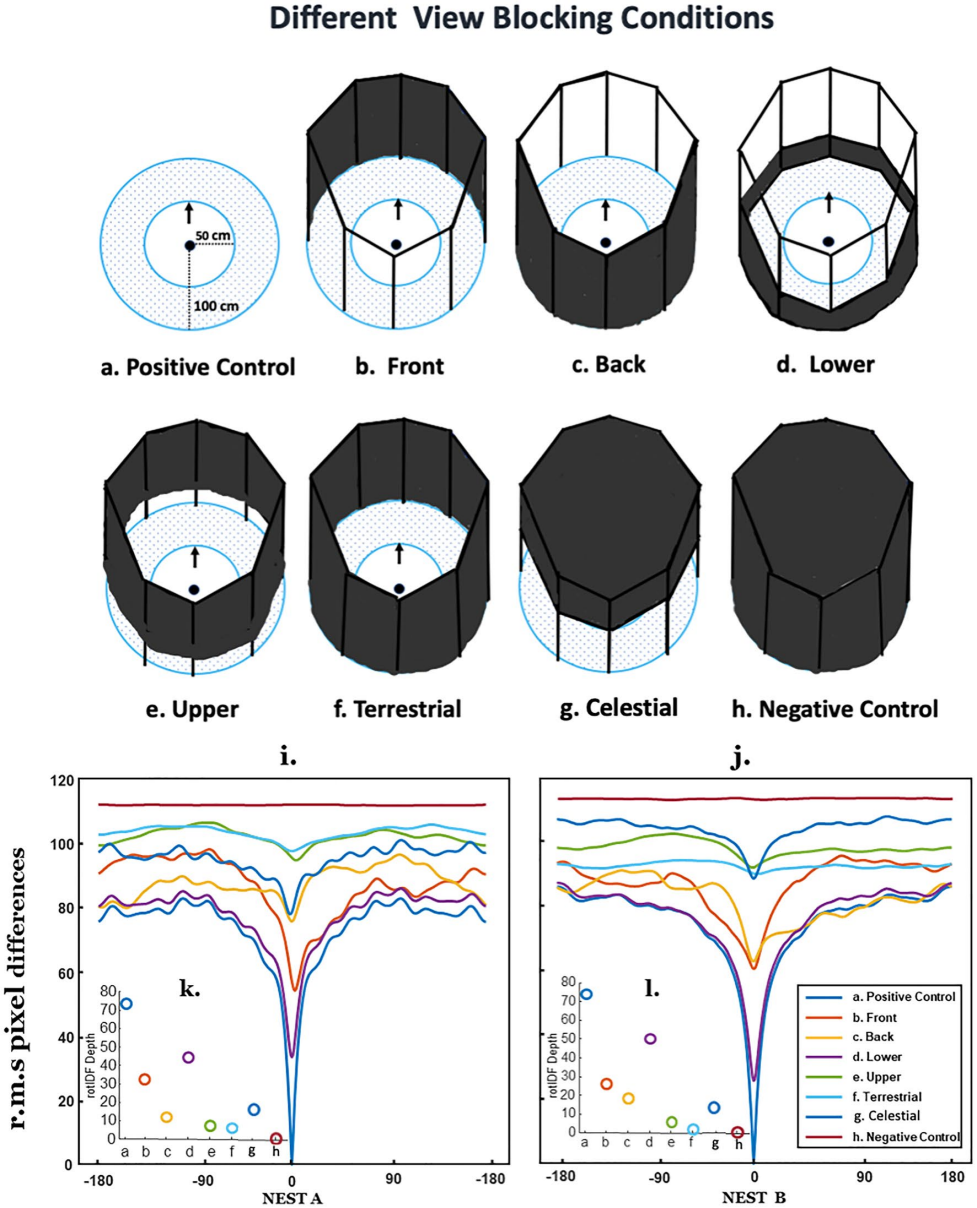
View regions blocked in different conditions (**a–h**), their rotational image difference functions (rotIDF) at each nest (**i** and **j**), and their rotIDF depth (**k** and **l**). In tests, foragers were released from the centre of a wooden platform, which was 50 cm in radius. The goniometer was divided into 24 sectors of 15° each. Ants were tested in eight different conditions: **a** Positive Control Condition: no view or cue blocking around the goniometer; **b** Front Condition: 50% of the view blocked in the front portion at 1 m radius from the goniometer centre; **c**. Back Condition: 50% of the view blocked at the back portion at 1 m radius from the goniometer centre; **d** Lower Condition: a 30 cm wall surrounding the goniometer (16.7° elevation); **e**. Upper Condition: a 90 cm black screen above a 30 cm gap; **f** Terrestrial Condition: a 120 cm wall around the goniometer; **g** Celestial Condition: a 50 cm screen blocking the upper portion and covering the canopy, leaving a 70 cm gap from the ground up; **h** Negative Control Condition: a 120 cm wall and roof top around the goniometer blocking the all of the visual cues. The bottom panel shows the rotation image difference function for each treatment, **i** at Nest A, and **j** at Nest B, each with an inset **k** and **l** which shows the rotIDF depth (depth = mean – minima)

## Experimental procedure

To conduct the experiments, we collected the outbound foragers in the evening twilight from the base of the foraging tree. Two foraging trees from Nest-A and one foraging tree from Nest-B were selected which were 6–7 m away from the forager’s nest. We cooled the foragers on ice for 2 min and painted them using Citadel Colour paint. After that, we allowed them 15 min to warm up and feed on a 20% honey–water solution. We then kept them in the laboratory overnight. Next morning, in daylight 7:00 am–10:30 am, we tested each ant once in a single condition. We set up a wooden platform (2 m × 2 m platform) 2 weeks before the experiment to allow adaptation, which we fixed so its centre was located ~ 4 m from the nest and ~ 2 m away the foraging tree. The wooden platform held a goniometer 50 cm in diameter with 24 sectors segregated into 15° bins. At the platform’s centre was a cup (15 mm wide and 30 mm deep) on to which we released the ants (See supplementary Figure S1).

### Filming technique

All releases were filmed with a tripod-mounted Go Pro Black Hero-8 (120 frames per second) camera suspended 700 mm above the goniometer. The camera’s field of view covered a 1000 mm × 1000 mm area centred on the release location. Ants were placed in the cup at the centre of the goniometer to start a test. A treatment was considered to begin once the ant crested the edge of the cup and to end when the ant crossed the 50 cm radius line. We filmed the ants using natural light in different treatments except in the negative control conditions where we used IR light because of darkness, light that does not affect any behavioural activities. We recorded each treatment for a maximum 12 min and had to discard the data for four ants in two different treatments which did not cross the boundary line during this time. We hand-drew the paths of each ant in each treatment based on these video recordings, and recorded scan number (defined in the Analysis subsection) and location using Quick Time Player.

### Experimental treatments

For this experiment we had one pair of controls and 3 pairs of treatments, each aimed to distinguish the importance of different portions of the ant’s panoramic view for visual navigation. Our three key comparisons were: Front vs. Back, Upper vs. Lower, and Celestial vs. Terrestrial, while our positive control showed baseline foraging behaviour, and our negative control showed what ants did when no visual information was available. In the *Positive Control* (*n* = 64, Fig. 1a), foragers were collected and tested without any changes in the panoramic view of the surrounding environment. For all of the rest of the blocking conditions, the distance between the centre of the releasing point and the blocking area around the goniometer was 1 m in radius. To investigate which side is most important, the front or the back, in the *Front* (*n* = 42, Fig. 1b), the front half of the skyline panorama (180° around the nest direction) was blocked by a semicircular black screen (120 cm in height), which was composed of light protective polyethylene (LPP) and black fabrics, while in the *Back* (*n* = 58, Fig. 1c), 180° of the semicircular panorama was blocked at the back side of the releasing point. To examine which elevations, either the lower or the upper, are more crucial for navigation, we conducted a test blocking the lower view of the surrounding panorama by making a 30 cm high wall around the goniometer centre (blocked the panorama up to 17 deg elevation), called the *Lower* (*n* = 54, Fig. 1d), and another test where the upper portion of the panorama was blocked with a 90 cm black screen above a 30 cm gap, called the *Upper* (*n* = 52, Fig. 1e). To examine whether terrestrial or celestial portions of the panorama are more important the ants’ navigational behaviour, in the *Terrestrial* (*n* = 62, Fig. 1f), we blocked all the terrestrial cues with a 120 cm high black screen completely around the goniometer centre, leaving the canopy open. In the *Celestial*** (***n* = 64, Fig. 1g), we blocked the canopy information by covering the roof (located 120 cm from the ground and 1 m in radius) and a 50 cm upper portion using a black screen. For the *Negative Control* (*n* = 54, Fig. 1h), we blocked all of the visual cue information by covering all of the surround and the top above the goniometer.

### Rotational image differences

We used a panoramic camera (RICOH THETA) to capture panoramic views of release points in all eight treatment conditions. The panoramic images were unwarped to rectangular panoramas; only their green channel was extracted, from 10° below the horizon to 90° above the horizon (total vertical FOV = 100°; total horizonal FOV = 360°), and low-pass filtered with a 3° Gaussian filter to match the visual acuity of *Myrmecia* ants (Narendra et al. 2011). We computed image similarities using the rotational image difference function (rotIDF), by comparing root mean square pixel differences for each 1° shift in pixels, between the Control Condition panorama and each of the eight views (for detailed methods see Zeil et al. 2003, Stürzl and Zeil 2007; Narendra et al. 2013; Narendra and Ramirez-Esquivel 2017; Murray and Zeil 2017). From these values we derived the mean and minima of these difference functions. We furthermore calculated the rotIDF depth for a given image pair as the rotIDF mean – rotIDF minima.

## Analysis

We used Graph-Click (www.arizona-software.ch/graph-click) to digitise the hand-drawn paths of foragers. A custom-written MATLAB program was used to plot the digitised paths of the foragers and calculate the path straightness of individual ants. The initial heading direction of foragers was analysed with circular statistics (Batschelet 1981) using the circular statistics software Oriana Version 4 (KOVACH Computing Service, UK). Rayleigh’s Tests were conducted on foragers’ initial orientation, testing if the distribution of headings was clustered or if data met the conditions of a uniform distribution (*p* > 0.05). A *V* test was conducted to test if the mean of the distribution of orientations was significantly oriented towards the nest. In each condition, we also computed the 95% confidence intervals of foragers’ headings.

The path straightness was calculated as the ratio between the total path length of individual foragers and the straight-line distance from the releasing point to point at which the forager crossed the 50 cm radius. The range of path straightness was 0 to 1. We calculated the duration (seconds) from the time a forager came out from the cup at the centre of the goniometer to the time that it crossed the 50 cm radius. When foragers stopped for more than 10 s in the same place, we considered it to be a resting period, and reduced this duration to 1 s. We recorded each time a forager stopped and scanned the environment by turning on the spot, and subsequently resuming navigation. These scans are saccadic movements which precede a pause in motion. We counted the number of scans during each trial and compared them between conditions. In the Positive Control Condition, more than 90% of scans were performed within a 15 cm radius of the centre of the goniometer, and before the ant chose a heading direction. We call this 15 cm zone the Start, and the remaining 35 cm radius we call the Route. To analyse the effect of our experimental conditions on path straightness, scans, and duration, we conducted Welch’s ANOVA tests, since they are suitable for data with heterogeneity of variance. Where we found significant differences, we then ran a Tukey post hoc test to compare between the Positive Control Condition and other conditions. For scans, we further compared scans at the Start vs. on the Route in our analyses.

## Results

In the Positive Control, after some quick scans near the centre of the goniometer, foragers of both nests walked paths that were straight and nest-directed, displaying little meandering. These characteristics together suggest competent navigation, whether due to scene familiarity, or access to vector and visual compass information. In the Front Condition, foragers reacted to the panoramic view change, with most ants meandering until they found their way around the blockage on either on the right or left side. In the Back Condition, displaced foragers looked around at the Start and also turned back to look along the route, thus decreasing path straightness, but their initial headings were mostly nest-oriented. In the Lower Condition, most of the foragers came out from the goniometer centre and performed scans, and a number of them showed meandering with scanning along their route, decreasing path straightness. In the Upper Condition, foragers performed more meandering near the goniometer centre and some of them performed a systematic search with scans along their route, but the ants’ initial headings were mostly nest-oriented. In the Terrestrial Condition, foragers performed some scans around the goniometer centre and then showed straight-line orientation in random directions. Conversely, when we blocked the canopy information but left the surrounding view open, most of the foragers showed the navigational pattern of the Positive Control ants, suggesting little impact of the missing canopy information. Finally, in the Negative Control Condition, when we blocked all of the visual information, foragers performed scans with meandering throughout their slow, circuitous journeys in scattered directions. Formal inferential statistical analyses support these impressions. Overall, we found that blocking the front view, the lower elevations, and the entire terrestrial surround had the biggest adverse effects on initial orientation. The initial heading orientations of ants in these conditions were uniformly distributed. With the front or the lower portion of the view blocked, ants also scanned more at the start and along the route and took longer to exit the goniometer. With the entire terrestrial surround blocked, the ants were, surprisingly to us, quick in exiting, with few scans, but in random directions.

### RotIDF depth and minima

RotIDF minima are visible in every treatment except for the Negative Control Condtion, which had no useful visual information (Fig. 1i–l**)**. While there are apparent differences between the two nests, the overall pattern is similar, with the Terrestrial and Upper Conditions having small rotIDF depths (2.37–7.24 r.m.s pd; Fig. 1k–l) and higher rotIDF means (92.43–103.58 r.m.s pd; Fig. 1i–j), whereas the Front, Back, and Lower Conditions have greater rotIDFdepths (11.85–50.68 r.m.s pd; Fig. 1k–l), and similar means (76.31–87.83 r.m.s pd; Fig. 1i–j) [Supplimentary Table 1]. The biggest difference between the two nests is in the Front and Back Conditions, which have similar rotIDF means (81.73–87.84 r.m.s pd; Fig. 1i–j) across both nests, but whose rotIDF depths diverge more at Nest A (front: 32.48, back: 11.85 r.m.s pd; Fig. 1k–l) than at Nest B (front: 26.43, back: 18.97 r.m.s pd; Fig. 1k–l) [Supplimentary Table 1]. Overall, from the rotIDFs, we would expect navigators to be able to find a heading in most treatments, except for the Terrestrial and Upper Conditions, where finding a minimum may prove difficult. Unsurprisingly, in the Negative Control Condition, with all useful visual cues blocked, the analysis suggests that navigation should be impossible.

### Initial homing direction

**Fig. 2:**
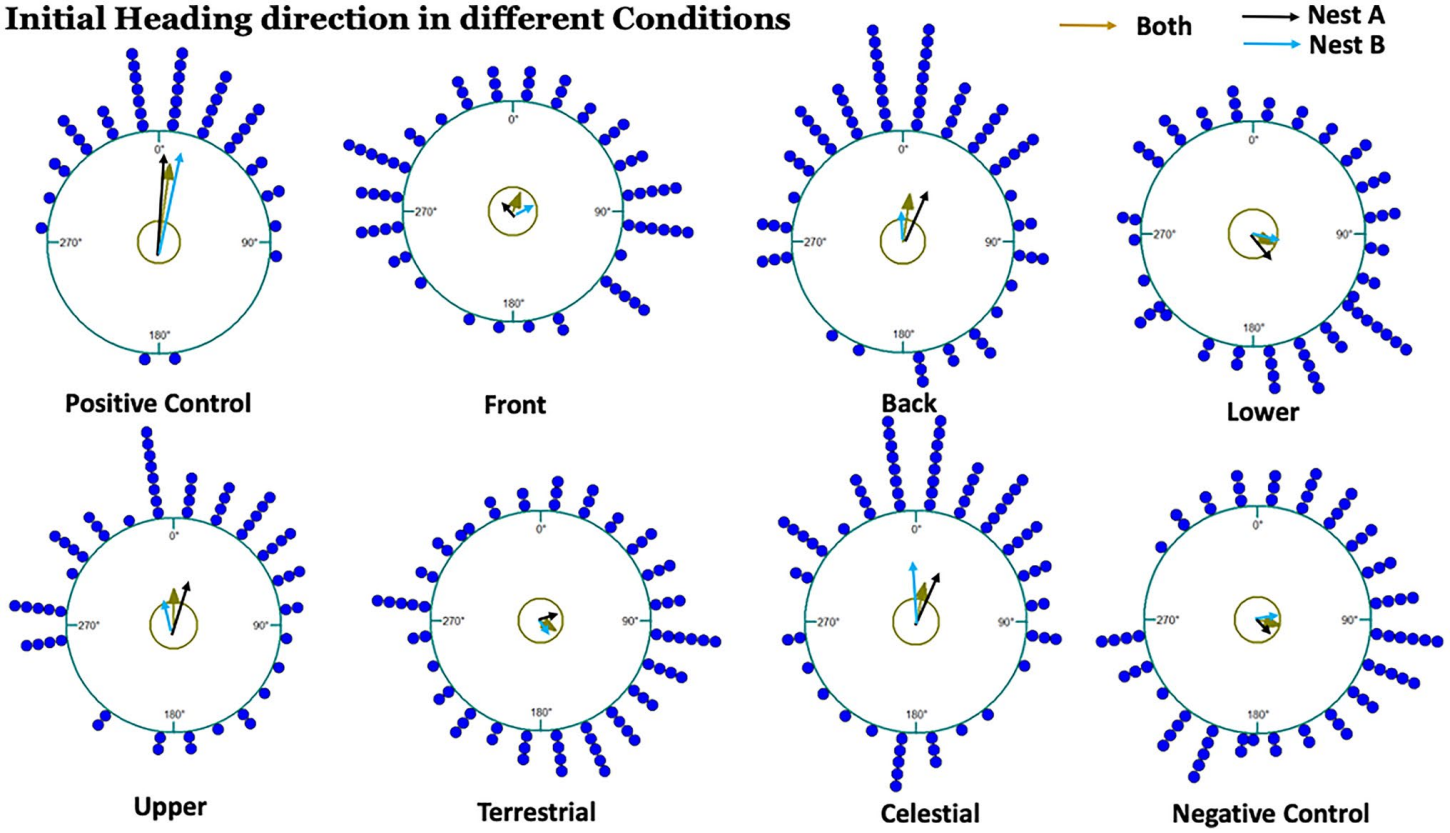
Circular histograms of initial headings of individual foragers of *M. midas* at 50 cm in the different conditions of their familiar routes. The histograms show orientation data in 15° bins, and the nest direction is indicated at 0°. The arrows in each histogram represent the length and the direction of the mean vector of foragers (see Table 2). The olive arrow indicates the combined mean vector of both nests whereas the black and sky colours denote Nest-A and Nest-B, respectively

**Fig. 3:**
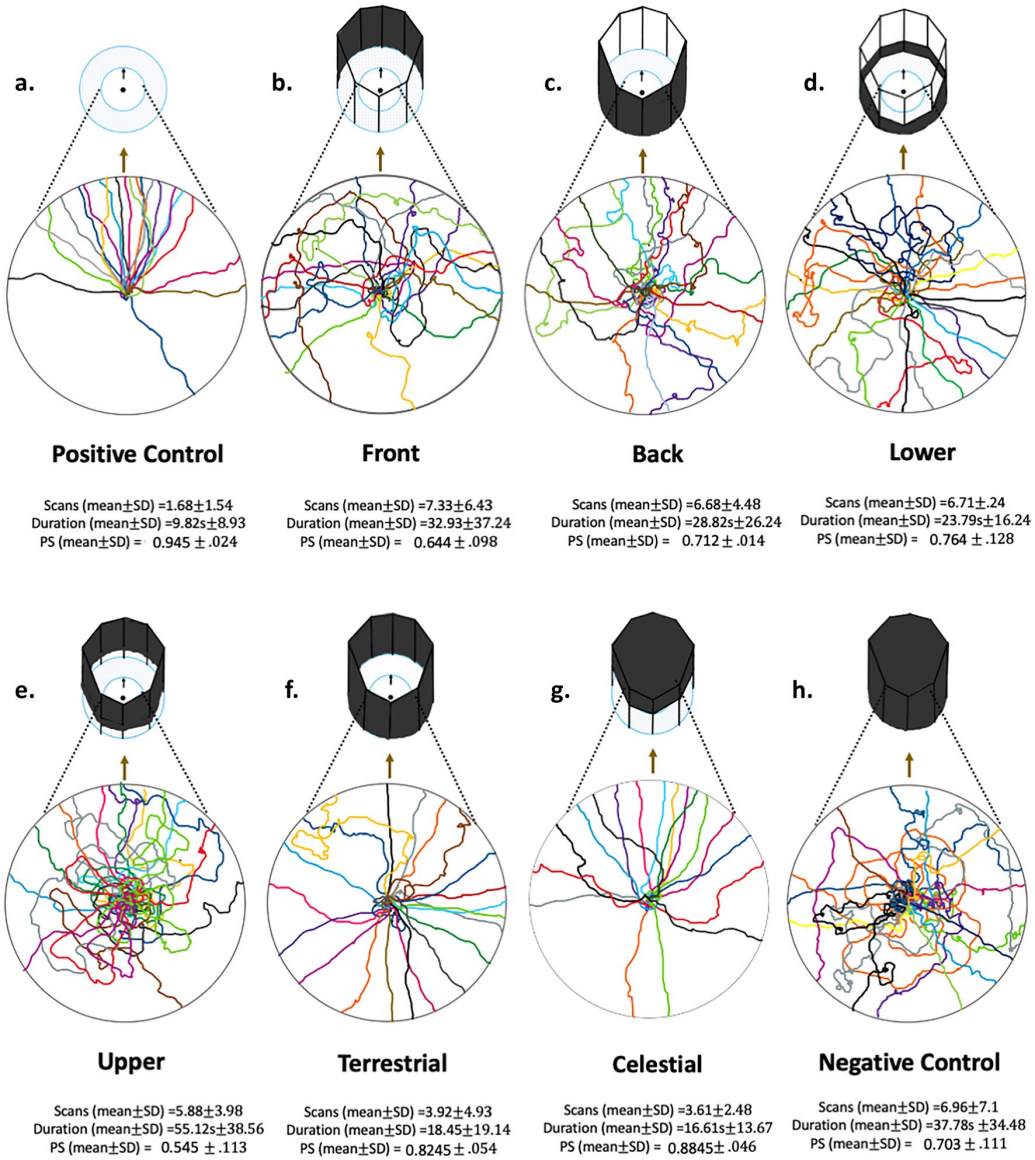
The paths of foragers in different experimental conditions on the goniometer. Ant path samples are selected randomly from all paths collected. The olive arrow indicates the nest direction, and the circle indicates the goniometer area (50 cm radius). In the Positive Control Condition (Fig. 3a), foragers were tested without any changes around the goniometer centre. The paths were randomly selected samples among all paths collected. The surrounding panoramic view and cues around the goniometer centre were changed in different ways (see details in Methods) in other conditions (Fig. 3b–h). Below each circle, the mean scans, durations, and Path Straightness (PS) with standard deviations are reported

In each of our key comparisons, where we blocked opposing portions of the panorama, foragers showed differences in their ability to find a nest-directed heading (Table 1; Table 2; Figs. 2 and 3). With none of the panorama blocked (Positive Control), foragers were non-uniformly distributed, with a mean heading not distinguishable from the nest direction at 50 cm (Rayleigh test, *p* < 0.0001; *V *test, *p* < 0.0001). In contrast, blocking the whole panorama (Negative Control) prevented the ants from finding the home direction; they instead headed in random orientations, forming a uniform distribution of initial headings with a mean significantly different to the nest orientation (Rayleigh test, *p* = 0.053; *V *test, *p* = 0.719). While foragers in one nest in the Back treatment formed non-uniform, nest-directed distributions (Rayleigh test, *p* < 0.0001; *V *test, *p* = 0.031; Table 1), when the Front was blocked, foragers at both nests showed no directedness, forming a uniform distribution of headings (Rayleigh test, *p* = 0.119; *V *test, *p* = 0.328; Table 1). In the Front treatment, ants showed a tendency towards a bimodal distribution (*D* = 0.059659, *p* = 0.08079) where some ants aimed towards the edge of barrier at 90°, but others headed towards the other edge of the barrier at 270° (Supplementary Fig. 2). Foragers also failed to find home-directed headings when the Lower panorama was blocked, showing uniform, non-nest-directed orientations (Rayleigh test, *p* = 0.075, *V*-test, *p* = 0.737; Table 1), but ants were nest-directed and non-uniform when the Upper panorama was blocked (Rayleigh test, *p* = 0.0081, *V *test, *p* = 0.0073; Table 1). Blocking the celestial or canopy panorama had no discernible adverse effect on homing orientation (Rayleigh test, *p* < 0.0001; *V *test, *p* < 0.0001; Table 1), whereas blocking the terrestrial panorama left foragers unable to find the nest-ward heading (Rayleigh test, *p* = 0.157; *V *test, *p* = 0.856; Table 1).

**Table 1:** Inferential statistics of all test conditions for both nests combined

Treatment	Distribution	Nest directed	Scan initial	Scan route	Duration	Straightness
Positive control	Non-Uniform	***p* < 0.0001**	***p* > 0.05**	***p* > 0.05**	***p* > 0.05**	***p* > 0.05**
Front	Uniform	p > 0.05	p < 0.05	p < 0.05	p < 0.05	*p* < 0.001
Back	Non-Uniform	***p* < 0.0001**	*p* < 0.05	*p* < 0.05	*p* < 0.05	*p* < 0.001
Lower	Uniform	*p* > 0.05	*p* < 0.05	*p* < 0.05	*p* < 0.05	*p* > 0.05
Upper	Non-Uniform	***p* < 0.0001**	*p* < 0.05	*p* < 0.05	*p* < 0.05	*p* < 0.001
Terrestrial	Uniform	*p* > 0.05	***p* > 0.05**	***p* > 0.05**	***p* > 0.05**	***p* > 0.05**
Celestial	Non-Uniform	***p* < 0.0001**	*p* < 0.05	***p* > 0.05**	***p* > 0.05**	***p* > 0.05**
Negative control	Uniform	*p* > 0.05	*p* < 0.05	***p* > 0.05**	*p* < 0.05	*p* < 0.001

The Distribution column was based on Rayleigh tests of the distribution of initial headings. The nest directed column was based on the *V* test for the nest direction. The other columns were based on post hoc tests against the positive control condition. Bold means similarity to the positive control condition

### Scanning

**Fig. 4:**
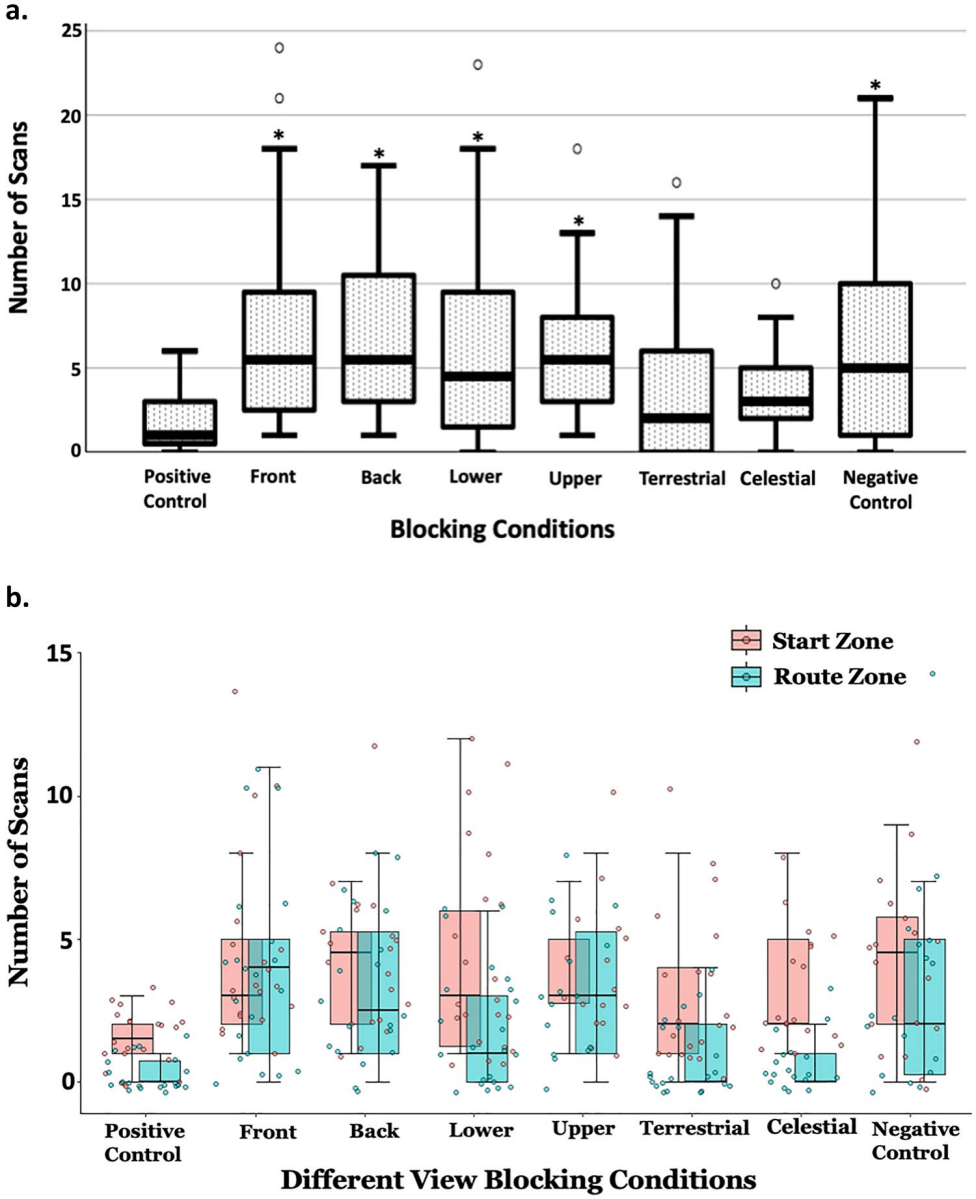
**a** The number of scans on the goniometer (50 cm radius) in different experimental conditions. A ‘*’ indicates significant differences in number of scans compared to the Positive Control Condition. The box plot indicates medians (solid black line), box margins (25th and 75th percentiles) and whiskers (5th and 95th percentiles). **b** The mean number of scans as a function of location on the goniometer in different view-blocking conditions. Same conventions for box plots

Foragers performed 1–2 scans in the first 50 cm of their return to the nest under normal (Positive Control) conditions, but most other treatments prompted an increase in the amount of initial scanning. These differences in scans across treatments were significant (Welch’s ANOVA: *F*_7, 78.56_ = 10.428, *p* < 0.001). Pairwise comparison to the Positive Control showed significant increases in total scans in the Front, Back, Lower, Upper and Visual Conditions (Tukey posts hoc tests *p* < 0.05; Fig. 4a), but no significant difference from the Positive Control in the Terrestrial and Celestial treatments (Terrestrial *p* = 0.716, Celestial: *p* = 0.855; Fig. 4a).

**Table 2:** Statistical outcomes of different conditions in Nest-A, Nest-B, and the two nests combined

Conditions	Mean vector µ (◦)	95% confidence interval			Rayleigh test			*V* test: direction 0°		
		Minus (◦)		Plus (◦)	*Z*	*p*		*V*	*p*	
Nest A										
Positive control	5.68	357.19		14.18	35.873	** < 0.0001**		0.869	** < 0.0001**	
Front	295.43	234.41		62.12	0.614	0.036		0.003	0.456	
Back	316.66	276.54		96.45	0.324	0.729		0.171	**0.004**	
Lower	137.63	35.67		239.67	0.598	0.554		− 0.103	0.789	
Upper	15.57	309.68		36.57	3.988	**0.019**		0.323	**0.003**	
Terrestrial	85.82	295.67		238.65	0.276	0.761		0.006	0.479	
Celestial	12.22	317.35		67.45	5.085	** < 0.0001**		0.441	** < 0.0001**	
Negative control	116.76	54.56		178.66	0.519	0.405		− 0.104	0.788	
Nest B										
Positive control	355.35	341.19		16.18	21.303	** < 0.0001**		0.688	** < 0.0001**	
Front	65.65	314.11		118.12	0.636	0.534		0.015	0.236	
Back	0.966	338.54		43.45	9.324	** < 0.0001**		0.567	** < 0.0001**	
Lower	104.32	41.77		166.67	1.598	0.204		− 0.054	0.619	
Upper	356.11	311.68		41.57	4.478	**0.008**		0.379	**0.0097**	
Terrestrial	136.98	98.22		178.65	3.079	0.119		− 0.264	0.789	
Celestial	7.59	333.15		42.58	5.851	** < 0.0001**		0.389	** < 0.0001**	
Negative control	147.85	83.56		202.66	4.802	**0.007**		− 0.328	0.966	
Combined										
Positive control	07.73	357.19		18.16	31.309	** < 0.0001**		0.701	** < 0.0001**	
Front condition	21.63	327.11		75.29	2.127	0.119		0.002	0.328	
Back	8.004	347.54		28.58	7.465	** < 0.0001**		0.431	** < 0.0001**	
Lower	115.78	66.45		164.86	2.597	0.075		− 0.088	0.737	
Upper	355.56	330.68		28.54	7.038	**0.0081**		0.342	**0.0073**	
Terrestrial	123.45	65.22		184.12	1.851	0.157		− 0.086	0.856	
Celestial	358.89	347.76		38.22	8.906	** < 0.0001**		0.389	** < 0.0001**	
Negative control	103.12	57.44		149.69	2.934	0.053		− 0.049	0.719	

Significant results are in bold

The patterns of scanning in the Start stage of travel and on the rest of the Route on the goniometer differed (Fig. 4b). At both stages of travel, variation among treatments was statistically significant (Start Welch’s ANOVA *F*_7, 78.14_ = 10.54, *p* < 0.001; Route: Welch’s ANOVA *F*_7,78_._14_ = 8.621, *p* < 0.001; Fig. 4b), but the pattern of post hoc comparisons differed between the Start and Route segments. At the Start, the number of initial scans was significantly higher than the Positive Control in all the treatments (Tukey post hoc: *p* < 0.05) except the terrestrial condition, which bordered on significance (Tukey post hoc *p* = 0.058; Fig. 4b). Compared to the Positive Control, the number of Route scans were significantly higher in the Front, Back, Lower, Upper and Negative Control (Tukey post hoc *p* < 0.05; Fig. 4b), but not in the Terrestrial (Tukey post hoc *p* = 0.238) or Celestial treatments (Tukey post hoc *p* = 0.445) [Fig. 4b].

### Duration on platform

**Fig. 5:**
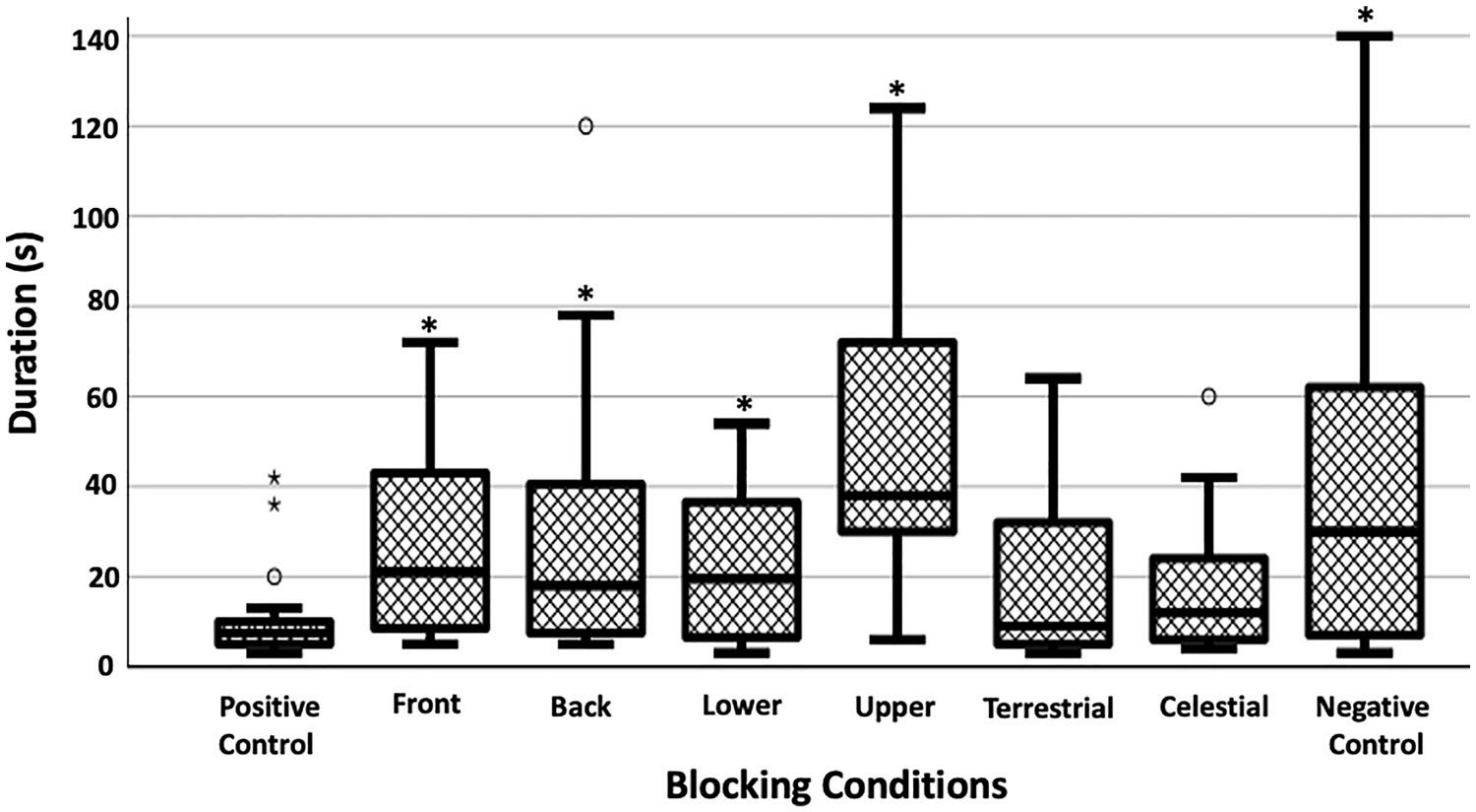
The duration of time that foragers took to pass 50 cm from the releasing point. Same conventions as in Fig. 4a

Except for Celestial and Terrestrial treatments, foragers spent significantly more time on the platform after emerging from the goniometer hole than they did when the whole panorama was visible (Positive Control) (Welch’s ANOVA *F*_7, 79.89_ = 8.621, *p* < 0.001). In the Positive Control, foragers left the platform quickly (*M* = 9.86 s), and in the Negative Control they took more than three times as long (*M* = 28.23s, Tukey post hoc *p* < 0.05; Fig. 5). Further Tukey post hoc tests revealed that compared to the Positive Control, the durations were significantly longer in the Front, Back, Lower, and Upper Conditions (*p* < 0.05), but not in the Terrestrial, and Celestial Conditions (*p* > 0.1; Fig. 5).

#### Path straightness

**Fig. 6:**
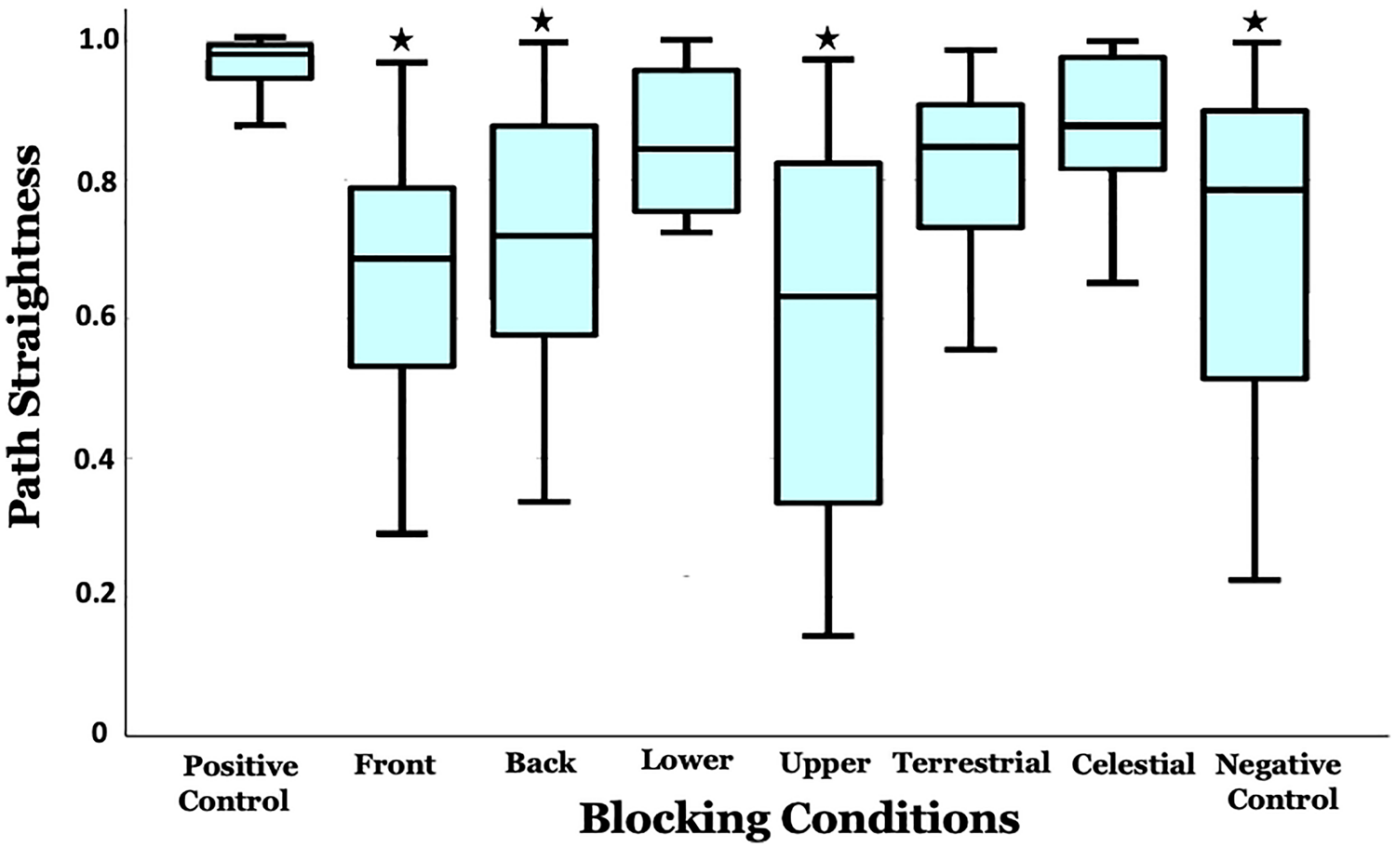
The Path Straightness of foragers in different experimental conditions. Maximum Path Straightness (a straight line) is 1. Same conventions as Fig. 4a

We found a large variation between treatments in the straightness of the plotted paths (Welch’s ANOVA, *F*_7,78.14_ = 23.812, *p* < 0.001; Figs. 3 and 6). Path Straightness of the Front, Back, Lower, Upper and Negative Control, were significantly different (Tukey, *p* < 0.05) from the Positive Control in the conditions, but Terrestrial, and Celestial were not significantly different from that control (Tukey, *p* > 0.1; Fig. 6).

## Discussion

Our experiment showed that some segments of the panoramic view are necessary for *M. midas* foragers’ navigation and while others are not, blocking them still impedes navigational performance. We blocked segments of the ants’ panoramic views in different directions and elevations in a familiar environment and measured their effect on rotIDFs and on foragers’ ability to find home. Consistent with the rotIDF results, under Positive Control conditions, these ants found a homing vector easily, and when the whole panorama was blocked (Negative Control and Terrestrial Conditions), they moved in random directions. Inconsistent with the rotIDF results, however, foragers were unable to reliably find a home vector when the front view or when the lower elevations were blocked; as such, we conclude that the front and lower segments are necessary for homeward initial heading in this species. Also inconsistent with image analysis, foragers were able to find a home vector when the canopy, the back view, and when the upper elevations were blocked; as such, we conclude that these segments are not necessary for correct initial heading. While the ability to navigate when the upper segment was blocked may imply that either the canopy, the lower segment, or the combination of the two cue sets was sufficient for correct initial heading, ants’ failure to orient homeward when only the canopy was visible (Terrestrial condition) leads us to conclude that the canopy is not sufficient for initial orientation. Given that if we ignore the role of the canopy, blocking the back segment is the inverse of blocking the front segment, we also conclude that having only the front view suffices for initial orientation.

We note what appears to be three broad categories of navigational behaviour; furthermore, within each category we note smaller within-category differences in speed and scanning. Conditions where foragers are well directed (Back, Upper, and Celestial) are similar to the Positive Control, but slower and with more scanning. Conditions where the ants are poorly directed (Lower and Front) are similar to the Negative Control, including being slower and having more scanning than the Positive Control, although we also note less meandering (more Path Straightness) for the Lower Condition (but see outliers in Fig. 6, which may be affecting statistical tests). A tendency towards a bi-modal distribution for the Front treatment suggests that a subset of the ants headed towards the either edge of the block at 90° or 270°, presumably to avoid the obstacle, circumvent it, and find open space. It is possible that some of these ants were negotiating a detour rather than seeking the rotIDF minimum as a strategy of navigation. The Terrestrial condition, discussed further below, is an exception where ants show fast, straight movement with no scans, and in random directions.

### What drives the importance of specific scene segments?

The discrepancy between predictions based on image analysis (rotIDFs) and the ants’ behaviours demands discussion. While the pixel-by-pixel analysis of rotIDFs is not meant as a model of how ants are processing visual information, it provides a proxy for how much useful visual information is found in any particular view for view matching. The discrepancies that we have found mean that some potentially useful visual information was not used by the bull ants (Front, Lower), but also that in other cases (Back, but especially Upper), ants are able to gain a heading with seemingly very little visual information. Such discrepancies suggest that ants are selective either about which portion of the panorama they pay attention to, or about which portion of the panorama they learn, or both. Selective attention and/or learning appears to be directed to the front part of the terrestrial panorama and the lower elevations of the terrestrial panorama. In the case of the Front condition, however, we have also considered, above, that some ants might be using a different strategy than view matching for navigation. They might be ignoring the direction of perceivable minimum of rotIDF and instead choosing to detour around the also visible block in front. Future research should address this issue.

An alternate interpretation of some of these results is that since ants are able to freely move, the proportion of the current view that is available grows or shrinks as they approach the edge. Such view transformations would mean that the vertical extent of the available panorama grows in the Upper condition, making homing easier, whereas it shrinks in the Lower condition, making homing harder. Such predictions are consistent with our data, as ants find a heading in the Upper but not Lower and Front conditions, which cause the greatest decreases in the extent of available views. Furthermore, ants move slowly and less straight in the Upper condition despite finding home, suggesting that although eventually successful, they have difficulty finding a heading. This third interpretation of the data along with view segment learning and view segment attention are all consistent with our findings and as such we suggest future experiments could be designed distinguish these phenomena, such as by fixing ants in place, or by moving the view blocker with the ant’s translation.

While our finding of the necessity of the front view for navigation in this species is consistent with findings in other species, the importance of the lower terrestrial panorama has not been explicitly tested before. The visual panorama is necessary for navigation in many hymenopterans (Knaden and Graham 2016; Freas and Schultheiss 2018), and while several experiments have blocked view segments (Graham and Cheng 2009; Reid et al. 2011; Schwarz et al. 2014; Freas et al. 2017a), few investigated how limiting access to visual information affects navigational ability. Like in *M. midas*, Reid et al. (2011) found that having even a small portion of the front view is important for *M. pyriformis* to find a foraging-tree heading. Similarly, they found that measures of navigational ease were reduced when some other view segments were blocked. Unlike the current study, however, they found differences between nests, causing them to conclude that navigational ability in response to view segment blocking is dependent on the visual composition of the local scene. While largely consistent, together these results suggest that this work should be expanded to more nests and more species, so that differences between local visual structure and its effect on individual behaviour can be disentangled from the effects of species-specific adaptations. Disentangling these contributions has implications for domains beyond biology, for the design of autonomous navigational systems, to ensure they are able to flexibly adapt their attention and learning to local conditions.

### Scanning for information or learning visual changes?

Blocking segments that were unnecessary for initial orientation still has adverse effects on the ease with which foragers found the home direction. With a full panoramic view, displaced foragers perform quick scans close to the release point, then move in a straight line and take little time to pass the 50 cm radius. But, like *M. pyriformis* and *M. bagoti* (Graham and Cheng 2009; Reid et al. 2011; Schwarz et al. 2014; Wystrach et al. 2014), when we blocked the front, back, lower or upper panoramic segments, our tested bull-ant foragers took longer to travel and performed more scanning than did the Positive Control group not only at the start of the trip, but throughout their route. In the Back Condition, foragers turned around and looked back towards their release point, beyond which the view was blocked. Scanning is interpreted as a response to navigational uncertainty—although we are not saying that the ant explicitly codes uncertainty—and as offering opportunities to learn views (Schwarz et al. 2014; Wystrach et al. 2019; Le Moel and Wystrach 2020; Murray et al. 2020; Islam et al. 2021a, b). From these results, we cannot infer to what extent scans are an attempt to lower current uncertainty by increasing information collection, versus learning for the future by storing views that have changed. It is possible our foragers were learning about the treatment-derived environmental changes for future navigation, which could explain the increased scanning even in conditions in which they were oriented in the home direction (Back and Upper Conditions). The exceptions (described below) are the celestial and terrestrial conditions. When the entire terrestrial surround is blocked, scanning does not increase, suggesting that the ants do not waste any time or effort seeking or trying to learn information that is not present. The lack of response to blocking the celestial panoramic features suggests that these features do not contain information sufficient to improve on that already provided by the terrestrial features.

### Celestial information and path integration

Celestial information is crucial for path integration, but since we find that the celestial view is neither necessary nor sufficient for *M. midas* to find home, this suggests that in this species, path integration is not a very important part of their homing strategy. As in Freas et al. (2017a), when the canopy view is all that remains, the foragers performed straight-line orientation in a random direction. These celestial cues were sufficient for this straight-line movement, and necessary in the absence of the terrestrial panorama, because when we blocked all visual information, including the celestial (Negative Control), foragers moved slowly and circuitously, stopping often. This straight-line orientation behaviour is reminiscent of ball-rolling dung beetles, which often pick a random orientation, and then rely primarily on celestial information, such as the moon and the Milky Way, to maintain that heading (Dacke et al. 2013, 2019, 2020). Since these nocturnal bull ants rely primarily on panoramic view to steer in the correct direction (Freas et al. 2017a; Freas and Cheng 2019; Islam et al. 2020, 2021a), they might be using a distinct navigational strategy similar to that of the dung beetles. When the bull ants have too little panoramic information to orient in the correct direction, they escape in any straight direction. We think this unexpected behaviour is worthy of further exploration of how such situation-driven behaviours help these animals to avoid falling prey to scenarios of temporary information paucity, so as to outperform state-of-the-art robots.

## Conclusion

From the current study we found that the front view, the lower elevations, and the terrestrial surround are more important for navigation in this species than are the canopy, the back view, and the higher elevations. These findings are somewhat inconsistent with the results of rotIDFs, which in isolation would suggest the lower segment is one of the least useful, while the upper segment is one of the most useful for navigation. This discrepancy suggests that ants have biases in which segments they learn or attend to, or that movement towards obstructions worsens their effect. Learning or attention biases could be the result of location-specific visual information, individual behaviour, or species-specific adaptations. We suggest that future experiments could determine the contribution of each of these factors, by testing a broader range of species and nest environments, while tracking individuals across their lifetime.

## Supplementary Information

Below is the link to the electronic supplementary material.

Supplementary material 1 (DOCX 9814.1 kb)
