# Supplementary material for: What view information is most important in the homeward navigation of an Australian bull ant, Myrmecia midas?
Source: J Comp Physiol A Neuroethol Sens Neural Behav Physiol. 2022 Sep 1;208(5-6):545–59. doi: 10.1007/s00359-022-01565-y (PMC9734209; doi:10.1007/s00359-022-01565-y)
Supplement: Supplementary file 1 — Supplementary material 1 (DOCX 9814.1 kb) [file 359_2022_1565_MOESM1_ESM.docx]

Supplementary Documents
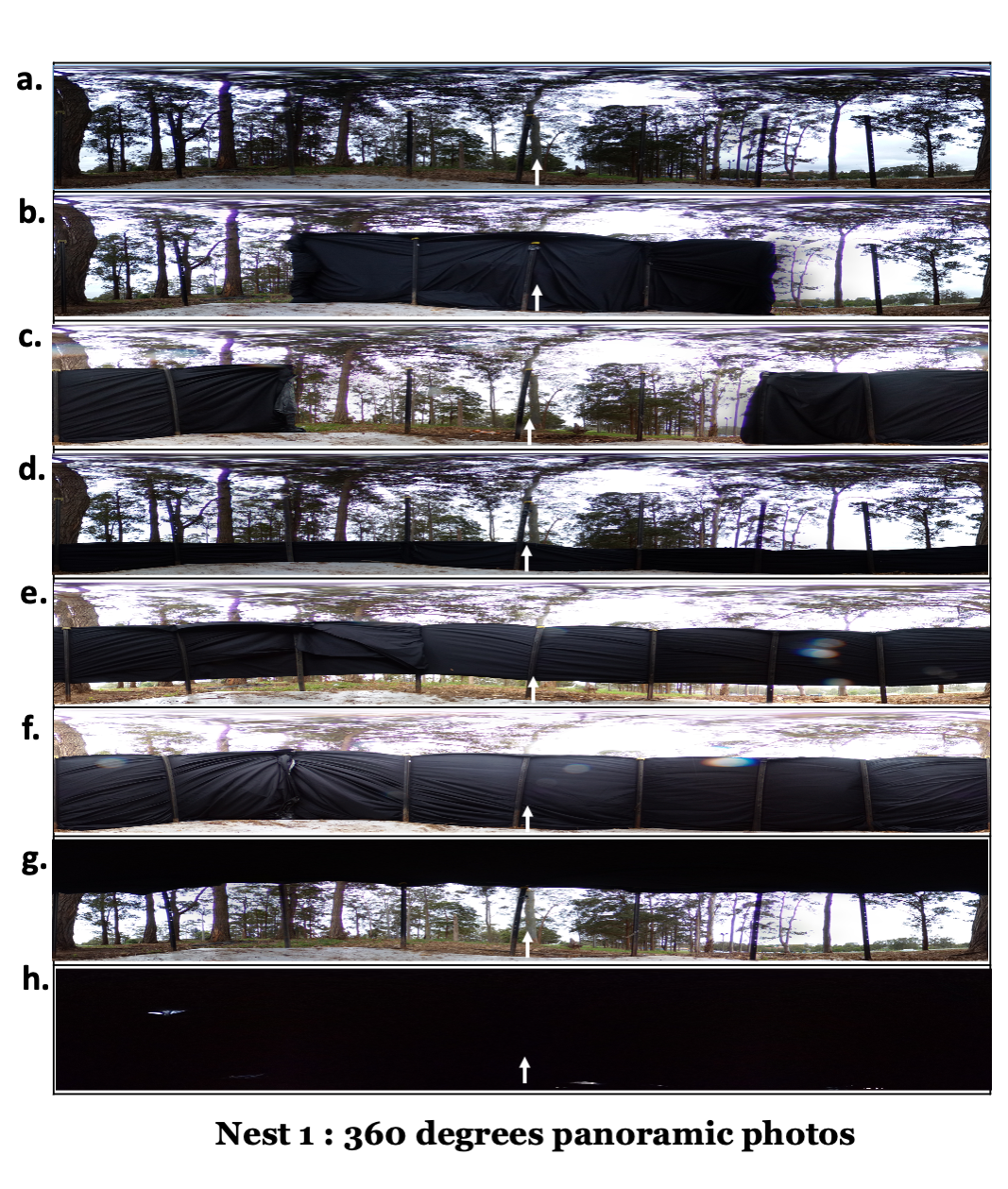


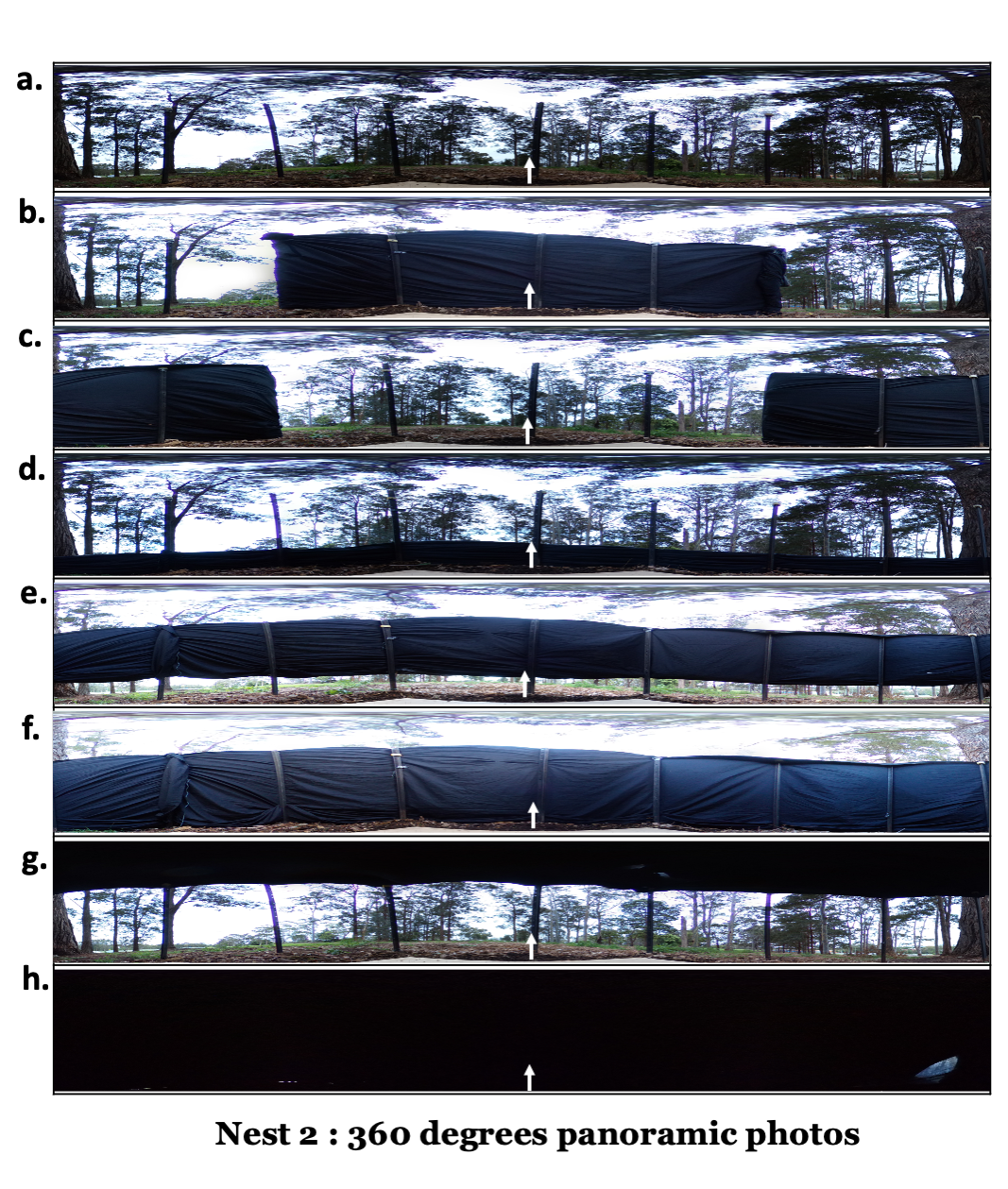


Supplementary Fig S1. Panoramic images at the release point of foragers in different view blocking conditions for Nest 1 and Nest 2: a. Positive Control b. Front, c. Back, d. Lower e. Upper g. Terrestrial h. Celestial & I. Negative Control. White arrows indicate the nest location.


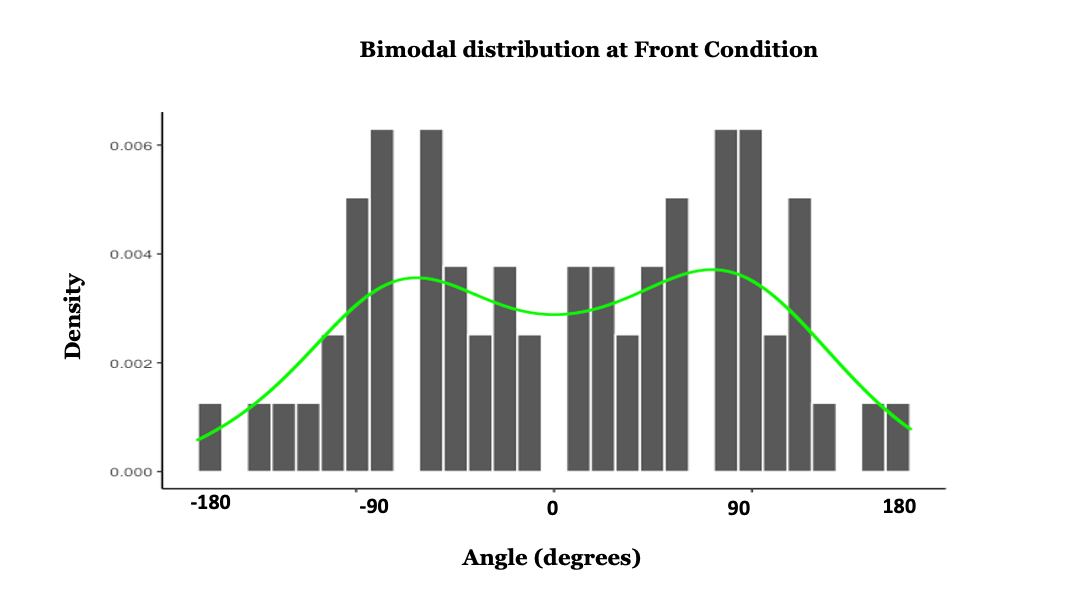


Fig S2. Bimodal distribution of ants heading in Front Condition (D = 0.059659, *p*-value = 0.08079) where some ants aimed towards the edge of barrier at +90°/–90. The fitted line is an atheoretical spline, there solely to illustrate the pattern of data.

### Table S1.

### Rot-IDF depth and minima for different view blocking conditions.
